# Neurological soft signs in early stage of schizophrenia associated with obsessive-compulsive disorder

**Published:** 2015

**Authors:** BE Focseneanu, I Dobrescu, G Marian, V Rusanu

**Affiliations:** * ”Titu Maiorescu” University, Bucharest, Romania; SanMed 2001 Medical Center, Bucharest, Romania; ** ”Carol Davila” University of Medicine and Pharmacy, Bucharest, Romania; “Prof. Dr. Al. Obregia” Clinical Psychiatric Hospital, Bucharest, Romania; *** ”Titu Maiorescu” University, Bucharest, Romania; “Prof. Dr. Al. Obregia” Clinical Psychiatric Hospital, Bucharest, Romania; **** Clinical Emergency Hospital, Bucharest, Romania

**Keywords:** neurological soft signs, schizophrenia, obsessive-compulsive symptoms

## Abstract

**Background.** Given that the obsessive-compulsive disorder (OCD) occurs with a much higher frequency in schizophrenia than in the general population, and, both schizophrenia and OCD are presumed to be neurodevelopmental disorders, the hypothesis of a distinct subtype of schizophrenia, the “schizo-obsessive” one, was raised.

**Aim.** Considering the neurological soft signs as neurobiological markers in schizophrenia, the aim of this study was to verify the hypothesis of the existence of this “schizo-obsessive” endophenotype of schizophrenia, by using the Neurological Evaluation Scale (NES) in patients with schizophrenia.

**Method. **The study was conducted in a transversal manner and consisted of the assessment of 64 patients with the maximum age of 26 years, who fulfilled the DSM IV-TR criteria of schizophrenia and/ or OCD, the assessment performed both from the social-demographic view, as well as neurologic, by means of the NES scale.

**Results.** Patients with schizophrenia and OCD proved to have, a significant family history from a static point of view, more loaded by affective disorders, but also by schizophrenia and OCD spectrum disorders, compared to pure schizophrenics. They also proved to have a significant higher educational level and a better occupational functioning than those schizophrenic patients without OCD, despite the similarity of the number of hospitalizations episodes or the disease duration to date. Ratings on the NES scale differentiate the group of patients with schizophrenia and OCD as having the highest scores on all subscales, scores much closer to those obtained by the group of patients with schizophrenia only, the only difference with statistical significance being recorded on the sequencing subscale of complex motor acts. The analysis of cluster through linear discriminant analysis allowed the classification of patients in the 3 groups with a probability of 89.06% and 76.56% for cross-validation.

**Discussion.** The results regarding neurological soft signs suggest that the presence of OCD in schizophrenic patients is due to peculiarities in fronto-basal ganglia circuits with possible origins in neurodevelopmental abnormalities. We considered that the early detection of neurological soft signs and their dynamic monitoring could provide useful information on the evolution of schizophrenia.

Future research should take into account larger groups of patients to investigate the relationship between neurological soft signs and brain neuroimaging data, as well as the results provided by neuropsychological investigations customed in this subgroup of schizophrenia.

## Introduction

Epidemiological studies have estimated that obsessive-compulsive disorder (OCD) occurs in a proportion of 12% of patients with schizophrenia, so more frequently than the general population [**1**], while the presence of obsessive-compulsive symptoms is estimated to occur in about 30% of cases of schizophrenia patients [**2**,**3**]. Studies suggested that this phenomenon would outline a particular clinical entity, the “schizo-obsessive” one, with a special neuropsychological profile and clinical course [**4**].

Both schizophrenia and OCD are considered neurodevelopmental disorders involving structural and functional abnormalities in frontal subcortical circuits [**5**,**6**]. Neurological soft signs in schizophrenic patients have been documented in various stages of the disease, including patients who are at risk of developing psychosis [**7**,**8**], patients with a therapeutically naive onset [**9**-**11**], but also to those in the chronic stage [**12**,**13**]. Also, these abnormalities have been shown to be relatively stable over time, without suffering any significant influence due to antipsychotic treatment [**9**,**10**].

Although, in 1980, Torrey [**14**] observed that neurological soft signs are associated with more severe and cronic forms of schizophrenia, discovery leading to the hypothesis that neurological soft signs are not static features in this disease, but varies during the couse of schizophrenia [**15**,**16**]. Although the presences of neurological soft signs in schizophrenia are well documented, this is not the case for OCD. However, there are few studies that not only reveal the presence of neurological soft signs in OCD [**17**-**19**], but some find these signs in patients with OCD comparable to those observed in patients with schizophrenia, including speed deficits and sequencing of motor acts deficits, suggestive of a fronto-subcortical dysfunction common to both pathologies [**20**]. Considering neurological soft signs in schizophrenia as neurobiological markers [**21**,**22**], this study aims to verify if there is a possibility of separation of the “schizo-obsessive” phenotype of pure schizophrenia.

For this purpose, we compared three groups of patients: 26 patients with schizophrenia without OCD, 17 schizophrenic and OCD patients and 21 patients with OCD, by using the NES neurological rating scale [**12**]. We were interested in following if there is a different pattern of neurological soft signs in the group of patients with schizophrenia and OCD compared to those patients with schizophrenia without OCD, or whether patients with schizophrenia and OCD showed higher rates of neurological soft signs probably due to an additive effect of neurological deficits generated by the presence of schizophrenia and OCD in the same patient.

## Method

**Subjects**

The study was conducted in “Prof. Dr. Al. Obregia” Psychiatric Clinical Hospital, between March 2014 and April 2015, the selection of patients being made according to a set of inclusion and exclusion criteria. The study group included 64 patients aged up to 26 years, diagnosed with schizophrenia (26 patients), schizophrenia and OCD (17 patients) or OCD (21 patients) according to DSM IV-TR [**23**], who underwent recent relapse (requiring hospitalization or corrective therapeutic intervention to avoid the confusion that would create in residual patients between obsessions and delusions with partial insight), but did not receive antipsychotic treatment for more than three weeks per episode; current (to limit the emergence of obsessive-compulsive symptoms under treatment). They also had a negative history of mental retardation, severe head trauma, another significant cerebral organic disease, significant neurological disorders, abuse/ addiction to substances in the last 6 months, other conditions that prevented neurological or psychometric assessment). The study was approved by the hospital’s Institutional Review Board and all the patients expressed their explicit consent for participating in the research trial.

The study group included patients of younger age, who were in the early phase of the disease, to limit the impact of factors such as disease duration on neurological evaluation results. The group of OCD patients was selected to test the hypothesis concerning the additive effect of neurological deficits related to schizophrenia and OCD, in the subgroup of patients with schizophrenia and OCD. We selected patients who met, at the same time, the diagnostic criteria for schizophrenia and schizophrenia with OCD and we excluded schizophrenics with mild obsessive-compulsive symptoms that did not meet the criteria for OCD according to DSM IV-TR, to accurately investigate possible differences between categories of patients with schizophrenia and to eliminate any interference that the presence of obsessive-compulsive symptoms would create in clinical and neurological evaluation. Patients with obsessive-compulsive symptoms strictly related to positive symptoms of schizophrenia (e.g. compulsive washing of hands due to imperative hallucinations) were also excluded.

**Clinical evaluation**

SCID-I/ P (the Structured Clinical Interview for DSM IV Axis I Disorders, Patient Edition [**24**] was used to diagnose schizophrenia and OCD. Neurological Evaluation Scale was applied to all patients to detect and score neurological soft signs, by the same clinician. This scale consisted of 26 items, rated between 0 and 2 (0 - normal, 1- some disruption; 2 - major disruption) which was divided into four subscales: the motor coordination that included riding in tandem walk, rapid alternations movements, finger/ thumb opposition, finger to nose test. The second subscale was sensory integration and included audiovisual integration, stereognosis, graphesthesia, extinction and right/ left confusion. The third subscale was the sequencing of complex motor acts and included fist-ring test, fist-edge-palm test, the Ozeretski test and the rhythm taping B test. The fourth subscale was that of “other” signs and included short-term memory, eye movements abnormalities (synkinesis, convergence, visual impersistence), frontal release signs (Romberg, adventitious overflow, tremor, memory, mirror movements, rhythm taping A test) and primitive reflexes (glabellar, snout, sucking, grasp). All patients filled in a questionnaire to collect socio-demographic data and personal and family psychiatric history, information being validated by data from medical records and/ or offered by a significant member of the family.

**Statistical analysis**

The information collected from patients was inserted into an Apache Open Office 4.1.0. database, statistical analyzes were performed by using R software (version 3.1.2.) and SAS-University Edition [**25**,**26**]. In terms of inferential statistical, the algorithm was for category variables either a χ2 test for proportions (if each category was present/ absent in at least 5 cases per sample) were used or a Fisher’s exact test (when the above mentioned condition was not met). Both tests were two-sided version. When possible, a confidence interval of 95% was also reported, which was either the value of scale differences, or the relative risk (risk ratio). For continuous variables, it was initially appreciated if distributions could be approximated by a normal distribution. If it was determined that distributions were close to normal distribution, an ANOVA test was performed. If the assumption of normality could not be sustained, a Kruskal-Wallis test was used. If multiple tests were required, procedures that protected against Type I errors (Bonferroni, Scheffe sampling without replacement) were used, procedures which were detailed for each test. If only two samples were analyzed, a two-sample Student’s test with Satterthwaite approximation for unequal versions was used. All tests were considered statistically significant at a level of sensitivity α = 0.05.

## Results 

The analysis of demographic and historical data is presented in the table below (**Table 1**). 

**Table 1:** Comparison of demographic and medical history characteristics of schizophrenia with and without OCD

Variables	Schizophrenia (n = 26) n/ mean (S.D.)	OCD (n = 21) n/ mean (S.D.)	Schizo phrenia + OCD (n = 17) n/ mean (S.D.)	Statistics	d.f.	P values	P values after correction (Bonferroni or sampling without replacement procedure)
Gender (M/ F)	5/ 21	12/ 9	8/ 9		2	0.022	
Place of birth (U/ R)	13/ 13	19/ 2	15/ 2		2	0.002	
Psychiatric family history							
affective	5	18	6		2	<0.0001	
schizophrenia	8	1	13		2	<0.0001	
OCD spectrum	2	8	9		2	0.004	
Number of hospitalizations	4.07 (2.81)	1.33 (0.73)	4.05 (3.34)	χ²=24.5636	2	<0.0001	0.0010a 0.0038b
Disease duration, months	44.76 (23.65)	87.90 (53.07)	58.00 (33.30)	χ²=8.8198	2	0.0122	0.0006a 0.0487b
DUD, months	44.76 (23.65)	87.90 (53.07)	58.00 (33.30)	χ²=8.8198	2	0.0122	0.0006a 0.0487b
Age at first treatment	20.15 (2.12)	20.66(1.82)	19.70 (2.56)	F=0.94	2	0.3972ANOVA	
Age upon first hospitalization	20.26 (2.23)	21.47(2.35)	19.82 (2.62)	F=2.55	2	0.0861ANOVA	
Age of the psychotic disorder onset	19.57 (2.46)		19.11 (2.54)	t=0.5852	35.4804	0.5621ANOVA	
Age of the obsessive-compulsive disorder onset		14.90(5.01)	12.81(4.99)	t=1.2750	36.4524	0.2104ANOVA	
Marital status							
Married	4	2	2	χ²=1.02	2	0.6015	
Consensual Relationship	2	6	3				
Single	20	13	10				
Divorcee	0	0	2				
Widower	0	0	0				
Educational level							
Unschooled	0	0	0	χ²=12.46	2	0.0020	0.003a 0.014c
Primary school	2	0	0				
Middle school	4	0	2				
High school	18	11	8				
Faculty	2	10	5				
Post-Graduate	0	0	2				
Occupational level							
Pupil	0	0	0	χ²=12.01	2	0.0025	0.0030a
Student	3	5	0				
Employed	3	9	4				
Unemployed	10	6	10				
Retired	1	0	0				
Handicap	9	1	3				
*DUD = duration untreated disorder, OCD = obsessive-compulsive disorder, S.D. = standard deviation, a, p<0.01 schizophrenia versus OCD; b, p<0.05 OCD versus schizophrenia+OCD; c, p<0.05 schizophrenia+OCD versus schizophrenia*							

The statistical analysis (One-way ANOVA) revealed no significant statistical differences in terms of age at the first hospitalization, age at the first treatment or between the number of episodes of illness or duration of the disease among the 3 groups of patients, nor in terms of age of onset of psychotic symptoms between pure schizophrenics and those who associate OCD (Two-Sample t test Welch two-sided) or the age of onset of the obsessive-compulsive symptoms between patients with OCD and those OCD associated with schizophrenia (Two-Sample Welch t test two-sided), although the onset of the obsessive-compulsive symptoms seems to be earlier in the comorbid group. There are significant differences in terms of the number of hospitalizations among patients with either schizophrenia or OCD (p = 0.0010, sensitivity level α = 1%), and between group with obsessive compulsive disorder and those who associate schizophrenia (p = 0.0038, sensitivity level α = 1%), but not between the two groups of schizophrenia (p> 0.05, sensitivity level α = 5%). The analysis of variables such as disease duration to study entry and duration until the first treatment of the disease detected statistically significant differences between the group of pure schizophrenia and the group of OCD (p = 0.0006, a level α = 1% sensitivity, respectively p <0.0001, sensitivity level α = 1%), and between the TOC and the comorbid groups (p = 0.0487, sensitivity level α = 5% and p <0.0001, sensitivity level α = 1%). There were no statistically significant differences between the 2 groups of schizophrenia with/ without associated OCD. The disease in the early years of the onset of schizophrenia appears to be similar in the 2 groups, independently of the presence of OCD symptoms, in terms of the frequency of relapses.

However, it was noted that in the group of patients with schizophrenia and OCD, the onset of the obsessive compulsive disorder precedes the onset of schizophrenia in 70.59% of cases, while in 29.41% of cases, the onset occurs relatively simultaneously, which is why obsessive-compulsive symptoms may be a prodromal feature in the onset of psychosis (according to the study Üçok et al. in 2011 [**27**]). In the group of schizophrenia, the onset occurred acute, reactive in 38.46% of cases, while in patients with schizophrenia and associated OCD, the reactive onset of psychotic symptoms occurred in 47.05% of the cases. The acute onset is considered one of the long-term positive outcome predictors in schizophrenia.

The research of family history (first-degree relatives) developed interesting data about patients with schizophrenia and associated OCD. The P-value <0.0001 obtained at the χ2 multicomparison test, as well as the fact that the estimates for the groups of schizophrenia and schizophrenia with OCD do not overlap, the same happening with the estimates for the group of schizophrenia and the group of OCD, allowing us to infer that there are statistically significant differences between the groups of schizophrenia and those of schizophrenia with OCD on the one hand, and between the groups of schizophrenia and OCD on the other hand. In terms of affective disorders in the family (p <0.01 level of sensitivity α = 1%), pure schizophrenia patients appear with affective disorders in a much lesser extent among relatives of the I/ II degree (19.23%) compared to those with OCD (85.71%) or to the schizophrenics associating (94, 12%). Also, this research showed that family history of schizophrenia is much loaded in patients with schizophrenia and OCD (76.47%), compared to those with schizophrenia (30,77%) or those with OCD (4.76%), value calculated at the χ2 test (p <0.0001), as well as the fact that none of the estimates for each of the 3 groups does not overlap others, allowing us to infer that there are differences with statistical significance regarding the antecedents of schizophrenia in the family (p <0.01 level of sensitivity α = 1%).

Research of the history of OCD spectrum in relatives of patients included in the study showed that by using the χ2 statistics, statistically significant differences between groups of patients with schizophrenia and those with schizophrenia and OCD, on the one hand, and between groups of patients with schizophrenia and those with OCD, on the other hand (p <0.01, sensitivity level α = 1%), the patients with pure schizophrenia have OCD spectrum disorders in their family, in a much lesser extent. The highest frequency was found in the group of patients with schizophrenia and comorbid OCD.

The statistics provided by the birthplace of patients in each group differentiated the patients with schizophrenia as having the birthplace distributed equally between urban and rural areas, from the patients with OCD and those with schizophrenia associated with OCD who were born in much higher proportion in urban areas. No differences regarding the marital status were found in the statistical analysis, but for the educational level, as a categorical variable, registered significant differences in Kruskal-Wallis analysis (p = 0.002) between the 3 groups. The multicomparison procedure (“post-hoc” comparison) was used to locate differences while being protected against Type I errors by the Bonferroni procedure, which revealed statistically significant differences between groups of people with schizophrenia and schizophrenia and OCD, on the one hand, and between patients with schizophrenia and those with OCD, on the other hand, the educational level being significantly lower in patients with pure schizophrenia (p <0.01, sensitivity level α = 1%, respectively p <0.05 level of sensitivity α = 5%). There were no statistically significant differences between groups of patients with OCD and those with schizophrenia associated with OCD, which allowed us to infer that patients with schizophrenia associating OCD have a higher preservation of the cognitive function, at least in young age, which can be a reason for escalating educational multistage compared to schizophrenic patients, possibly because of better cognitive functioning and recovery between episodes. The confirmation of this hypothesis was also strengthened by the research on occupational levels in the 3 groups of patients. The Bonferroni correction in the occupational level analysis revealed statistically significant differences between the group of patients with schizophrenia and the OCD patients (p <0.01, sensitivity level α = 1%), the group of patients with schizophrenia associated with OCD having an occupational level closer to that of the patients with OCD. 

The results obtained from the neurological evaluation by using the NES scale are shown in the table below (**Table 2**). 

**Table 2:** Comparison of NES scores of schizophrenia with and without OCD, and OCD

Variables	Schizophrenia (n = 26) n/ mean (S.D.)	OCD (n = 21) n/ mean (S.D.)	Schizo phrenia + OCD (n = 17) n/ mean (S.D.)	Statistics	d.f.	P values	P values after correction (BonferronI, sampling without replacement or Scheffe procedures)
NES Total	6.65 (5.04)	4.57 (4.58)	10.00 (4.89)	χ²=11.37	2	0.0034	-34.83 to -5.86b,d 0.02715c,e
NES motor coordination	0.80 (0.98)	0.52 (0.67)	1.05 (1.29)	χ²=1.23	2	0.5406	
NES sensory integration	1.50 (1.55)	1.09 (1.48)	2.23 (1.32)	χ²=6.79	2	0.0336	-29.43 to -1.18b,d
NES Sequencing on complex motor acts	2.34 (1.85)	1.66 (2.53)	4.64 (2.08)	F=13.30	2	< 0.00001ANOVA	-4.48 to -1.47b,d -3.74 to -0.86c,d
NES “other”	2.00 (1.93)	1.33 (1.33)	2.05 (1.98)	χ²=3.63	2	0.1627	
*OCD = Obsessive-Compulsive Disorder, NES = Neurological Evaluation Scale, S.D. = standard deviation, a, p<0.01 schizophrenia versus OCD; b, p<0.05 OCD versus schizophrenia+OCD; c, p<0.05 schizophrenia+OCD versus schizophrenia; d, IC95% for the difference arithmetic mean rank; e, Wilcoxon rank-sum test*							

Regarding the NES total score, the Kruskal-Wallis test revealed significant statistical differences between the 3 groups of patients (p=0.0034) and the procedure of multicomparison (“post-hoc” comparison) protected against type I errors through the Bonferroni procedure, it locates significant statistical differences between groups of patients with schizophrenia and OCD and those with OCD, but not between those with schizophrenia and schizophrenia associated with OCD, patients with schizophrenia and OCD obtaining the highest scores, followed by those with pure schizophrenia, the lowest scores being recorded in the group with OCD. Because the theme of the work and the fact that the upper edge of IC95% value for the comparison between groups of patients with schizophrenia and those with schizophrenia and OCD is close to 0, it was decided to carry out a separate Wilcox on comparison test (rank-sum), which proved that there is a significant statistical difference between the two groups (p=0.02715). Note that the results of the multicomparison test and the Wilcoxon test are contradictory. As a result, we believe that further studies are needed to make an in depth investigation so as to see if there are differences with statistical significance regarding NES total score between patients with schizophrenia and those with schizophrenia and OCD.

On the motor coordination subscale, the Kruskal-Wallis test showed no statistically significant differences between the 3 groups of patients (p=0,5406). Significant differences were observed between all groups, but on the sensory integration subscale (p=0.0336), and after Bonferroni correction procedure, significant statistical differences were revealed between groups of patients with OCD and schizophrenia associated with OCD, but also between groups of patients with schizophrenia and schizophrenia associated with OCD, patients with schizophrenia and OCD hovering, in terms of severity, on the first place, followed by pure schizophrenics, the lowest scores being recorded in patients with OCD. Following the Bonferroni correction procedure it could be seen that there are significant statistical differences between groups of patients with OCD and schizophrenia associated with OCD, but not between patients with schizophrenia and those with OCD, or between those with schizophrenia and schizophrenia associating OCD. Thus, we afforded to infer that, in terms of sensory integration, patients with schizophrenia and OCD are closer to the schizophrenia spectrum than those with OCD. 

The sequencing subscale of complex motor acts highlighted the group of patients with schizophrenia and OCD as having the highest scores. The analysis of the one-way variance (one-way ANOVA) allowed us to infer only that the 3 samples come from different populations (p<0,00001). A procedure of multicomparison was used to locate the differences by using statistics calculated by the ANOVA test (“post-hoc” comparison) protected against type I errors by using the S procedure described by Scheffe. Following the application of this procedure, statistically significant differences were observed between groups of patients with schizophrenia and schizophrenia associated with OCD (p <0.01) and between groups of patients with OCD and schizophrenia associated with OCD (p <0.01). There were no significant statistical differences between the groups of patients with schizophrenia and those with OCD (p = 0.45). On the “other” NES subscale, the scores obtained by the patients in the 3 groups were close, no significant statistical differences being identified at the Kruskal-Wallis test. There were no correlations between NES scores and other variables considered in the analysis.

In an attempt to find a model to classify patients according to the group they belong to, be in the schizophrenia group, either in the TOC, or in the schizo-obsessive linear discriminant analysis [**28**] was used to find out which variables can discriminate between two or more classes. Predictors like NES score, age, number of hospitalizations, duration of illness, were used as continuous variables, and family history of affective, obsessive or psychotic disorders, sex, origin, educational level were used as ordinal or categorical variables.

**Fig. 1:**
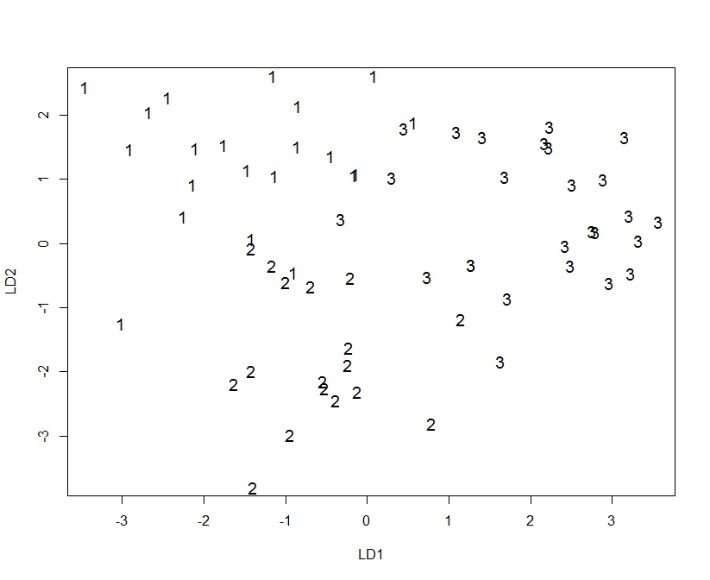
The LDA model in study group LD1 = linear discriminant 1, LD2 = linear discriminant 2, 1-OCD, 2-Schizophrenia+OCD, 3-Schizophrenia

The proportion of variability in the sample of patients was explained by one discriminant value of 0.630 and discriminant 2 with a value of 0.370. What should be noted is that the variability in the sample of patients is entirely explained entirely by the two discriminating elements (see **Fig. 1**).

The model correctly classified 57 patients and 7 patients wrong (the success rate was of 89.06%). To validate the model, a procedure known as Leave-one-out cross validation – LOOCV, was used. The cross-validation was correctly classified in 49 patients and 15 patients wrong (the rate was of 76.56%). Although the model has quite a high reliability, even through the validation procedure fell by 12.5% of its accuracy, it must be interpreted in light of the fact that the clinical picture may change in dynamic causing dragging and integration into a different nosological category than the present. The above data support the hypothesis of a particular endophenotip of schizophrenia, schizo-obsessive one, the share of genetic factors having a role in model determinism, as well as the presence of neurological soft signs, behaving like neurobiological and genetic markers in schizophrenia associating obsessive-compulsive symptoms. 

## Discussions

The major breakthrough of this cross-sectional study is that the soft neurological signs rated through the NES scale differentiated patients with schizophrenia associated with OCD from those with schizophrenia, particularly locating differences in the sequencing subscale of complex motor acts. Therefore, it can be concluded that poorer performance on tests of this subscale may be due to dysfunction in frontal-basal ganglia circuit.

There were no differences in the motor coordination subscale between the 3 groups of patients, contrary to studies [**29**,**30**] which showed significantly higher scores of neurological soft signs in this subscale, and in the sensory integration subscale, showed no statistically significant differences between the 2 groups of patients with schizophrenia with/ without OCD, but did show significant statistical differences between schizophrenia associated with OCD and the group of OCD patients. The result was similar to the one provided by Tumkaya et al. in 2012 [**31**] who found that patients with schizophrenia have performed worse than those with OCD, at all NES subscales, except for the sensory integration. Similar results were provided by the study of Bolton et al. in 1998 [**30**]. Therefore, one can imagine that patients with schizophrenia and OCD are closer, in terms of neurological profile, to those with schizophrenia.

It was also proved that no demographic variable such as the age of onset of psychotic or obsessive-compulsive symptoms or the number of hospitalizations, duration of the disease, influenced the pattern of neurological soft signs. Most research conducted showed no effect related to the gender on the NES [**32**]. The effect of the patients’ age at examination on NES remains unclear. While data on the correlation between older age and higher NES score are contradictory [**32**], most of these studies are cross-sectional in design and do not delineate very clearly factors that may co-vary with the age, such as the disease duration. We believe that studies are needed to analyze the impact of age and other potential mediators such as gender and educational level or the patient’s cognitive reserve on neurological soft signs in schizophrenia [**33**].

This research may suggest that the association of schizophrenia with OCD is more than just a comorbidity between two mental disorders that combine the neurological deficits of each one of them. It seems that this association, at least in terms of neurological deficits displayed, is part of the spectrum of schizophrenic disorders.

Rating neurologic soft signs on large lots of patients with schizophrenia, schizophrenia associated with OCD, with neuro-cognitive assessments and brain imaging, can help detect more specific neurological deficits in patients with schizophrenia associated with OCD.

In terms of demographic variables in the study, we were surprised by the reactive acute start of schizophrenia in patients who also presented OCD, in about 50% of cases, knowing that this type of onset is a favourable prognostic factor in the development of schizophrenia. This might also explain the significantly higher educational level achieved by patients with schizophrenia and OCD versus those with pure schizophrenia, despite the similarities in age of onset of psychotic symptoms or the number of hospitalizations to date in the 2 groups. Another explanation was offered by Poyurovsky’s study [**34**], who found that patients with schizophrenia who were at the first episode and who associated OCD had a less formal disorder in thought and less flat affection. The authors suggested that OCD, at least in the early stage of the disease, might have a protective effect in the development of schizophrenia. In another study, patients diagnosed with both schizophrenia and with OCD, had fewer negative symptoms and higher functionality scores than those diagnosed only with schizophrenia [**35**].

Also, the early detection of neurological soft signs and their quantification can provide useful information on the evolution of schizophrenia, since the meta-analysis conducted by Bachmann in 2014 [**36**] confirmed that the neurological soft sign scores decrease as they get psychopathological symptoms remission in schizophrenia and that this effect is more pronounced in patients who achieve remission than in those who do not achieve remission.

From the clinical perspective, the changes in the dynamics of soft neurological signs may be useful in monitoring the progression of schizophrenia and in identifying patients who are at an increased risk of developing schizophrenia and also have a much severe chronic illness. Because the neurological soft signs involve alterations in sensory and motor function as well as motor coordination and sequencing of complex motor acts, we believe that their deeper research contributes to the development and optimization of physical training programs, which already represent elements of routine therapeutic management in schizophrenia [**36**].

**Limitation**

The main limitation of this study is the small number of patients in all 3 groups, especially in the group of patients with schizophrenia concomitant with OCD, which sometimes provided contradictory results, statistically speaking, when locating differences between groups. Studies should be conducted on preliminary quantified samples to increase the power of discriminatory analysis from the statistical point of view.

Another important limitation was represented by the lack of impartiality of the clinician/ lack of blinding of the clinician that could lead to bias/ possible errors during neurological evaluations.

## Conclusion

The results of this study indicate that neurological soft signs could discriminate between patients with schizophrenia associating OCD and those with pure schizophrenia, in terms of location and severity, especially on the subscale sequencing complex motor acts and may have a value of endophenotype in researching specific etiological mechanisms underlying the “schizo-obsessive” group of schizophrenia.

**Disclosures**

None of the authors have any conflict of interest regarding this manuscript.

**Acknowledgements**

We are grateful to Dan Popescu, MD, for his support in data analysis.
